# The coccosphere of the heavy calcifying coccolithophore *Coccolithus braarudii* provides defense against bacteria

**DOI:** 10.1038/s42003-026-10585-2

**Published:** 2026-07-03

**Authors:** Sophie T. Zweifel, Richard J. Henshaw, Roberto Pioli, Clara Martínez-Pérez, Uria Alcolombri, Zachary Landry, Roman Stocker

**Affiliations:** 1 Institute of Environmental Engineering, Department of Civil, Environmental and Geomatic Engineering, ETH Zurich, Zurich, Switzerland; 2 Limnological Research Station, Department of Hydrology, University of Bayreuth, Bayreuth, Germany; 3 A. Silberman Institute of Life Sciences, Faculty of Sciences, The Hebrew University of Jerusalem, Jerusalem, Israel; 4 Department of Biological Sciences, University of Southern California, Los Angeles, CA USA

**Keywords:** Microbial ecology, Water microbiology, Microbial ecology

## Abstract

Coccolithophores produce 40–60% of marine calcium carbonate, largely through biomineralization of plates which encase cells in a ‘coccosphere’. Despite the importance of coccolithophore calcification in ocean biogeochemistry, its function remains unresolved. A hypothesis suggesting it acts as a physical deterrent has been investigated in grazers and viruses, but not in bacteria. Here we show bacterial pathogenicity in heavily-calcified *C. braarudii* treated with *Gephyrocapsa huxleyi* bacterial-pathogen, *Phaeobacter inhibens*, is only observed with decalcified algae, leading to algal-cell death within as little as 15 hours. Decalcified algal cell mortality is *P. inhibens*-specific and likely requires close proximity, since treatment with bacterial supernatant or growth-inhibiting concentrations of indole-3-acetic acid shows no detrimental effect. Additionally, scanning electron microscopy shows visible bacterial attachment only on decalcified *C. braarudii*. These findings provide the first experimental evidence that the coccosphere can act as a barrier against specific bacteria, highlighting its defensive role in coccolithophores.

## Abstract (web summary)

Decalcified cells of the coccolithophore *Coccolithus braarudii* collapse iupon exposure to *P. inhibens*, whereas calcified cells remain unaffected. This study provides the first direct evidence of the ecological hypothesis that calcification serves as a microbial defense strategy in coccolithophores.

## Introduction

Phytoplankton are responsible for an estimated 50% of global primary production^1,2^, with nearly 10% of their collective biomass comprised of a group of calcifying microalgae called coccolithophores^3,4^. Beyond their important role in the organic carbon pump through photosynthesis^5^, coccolithophores mineralize an estimated 40-60% of marine calcium carbonate^3,6,7^, thus playing a major role in the inorganic carbon cycle. This ultimately results in the annual sequestration of over a billion tons of calcium carbonate into the deep ocean when these organisms sink upon death^8^.

Coccolithophores mineralize calcium carbonate (CaCO₃) into intricate plates known as coccoliths, which are extruded and arranged—typically in one or more interlocking layers—forming a complete or near-complete shell called the coccosphere^9,10^. This mineralized covering makes coccolithophores a key model system for biomineralization^9–14^. Significant work has been dedicated towards understanding the intracellular mechanisms that govern this process^15–17^ and how environmental conditions affect calcification^6,13,18^. Calcification of the cell body can consume as much as 30% of a cell’s total photosynthetic energy budget^9^. Thus, we can expect that the shell must provide a considerable ecological benefit, yet this benefit remains unclear. Understanding the ‘why’ of calcification is a major open question in microbial oceanography^9^, whose answer will help to better predict how changes to calcification, due to environmental pressures such as ocean acidification, might ultimately influence these globally important organisms.

The function of the coccosphere has been explored through a range of hypotheses and experimental studies, revealing that its function likely differs across coccolithophore species^9^. The deep-dwelling coccolithophore *Schyphosphaera apsteinii* mineralizes a higher number of trumpet-like coccoliths in low light conditions: its coccoliths have been proposed to improve light collection^19^. In contrast, shallow-dwelling species, such as *Gephyrocapsa huxleyi* (formerly *Emiliania huxleyi*^20^), have flatter coccoliths that can serve as photoprotection^21^. The process of calcification through precipitation of CaCO_3_ has also been shown to shuttle CO_2_ to the immediate surroundings of the cell, which might enhance photosynthesis^22–24^. Other hypotheses have focused on the role of the coccosphere in cell interactions, as a protective barrier against grazing by zooplankton or infection by bacteria or viruses^9^. Although predation rates by dinoflagellates do not differ between calcified, non-calcifying, and artificially decalcified *G. huxleyi* cells^25^, dinoflagellates that consume calcified cells have been found to grow more slowly^26^. A further function of the coccosphere could be as a defensive “shell” or mechanical barrier to protect the cell from attack from viruses and pathogens. Investigation into viral infection of *G. huxleyi* has shown that the coccosphere offers only limited protection against viruses^27^ and in two studies the coccosphere was found to even enhance infection^28,29^. Given that marine viruses can be as small as 20 nm in diameter, while the smallest reported gaps in the *G. huxleyi* coccosphere are 200 nm in size^9,30^, it is not unexpected that the coccosphere only provides limited (if any) protection against viral infection^9^. In contrast, the physical structure of the coccosphere would be almost impenetrable for bacteria, yet the hypothesis that the coccosphere serves as protection against bacteria has to date remained untested.

Here, we investigate the role of the coccosphere in protection from bacterial attack with the coccolithophore *Coccolithus braarudii*: a heavily calcified coccolithophore species and the dominant calcifier in subpolar regions such as the North Atlantic^31,32^. The choice of *C. braarudii* over the model organism *G. huxleyi* has two main reasons. Firstly, unlike *G. huxleyi*, which can persist in a non-calcifying diploid state, *C. braarudii* exhibits a strict dependence on its coccosphere—its cell cycle arrests when calcification is inhibited^10,32,33^. Secondly, the extensively characterized “Jekyll-Hyde” interaction between *G. huxleyi* and bacterium *Phaeobacter inhibens*, whereby *P. inhibens* initially releases growth-promoting concentrations of indole-3-acetic acid (IAA), but switches to produce both growth-inhibiting concentrations of IAA and algicidal molecules once the algae senesce, occurs regardless of *G. huxleyi*’s calcification state. The *P.* *inhibens-*mediated culture collapse has been reported in both calcifying and non-calcifying *G. huxleyi* strains^34,35^.

In this work, we show that the coccosphere of *C. braarudii* protects the cell from opportunistic bacterial attack. When exposed to coccolithophore-associated bacteria, including *P. inhibens*, artificially decalcified cultures of diploid *C. braarudii* rapidly collapse, whereas calcified cultures remain unaffected. This response is independent of the method of decalcification. We show this response is strain-specific, with a panel of bacterial isolates from a previous mesocosm bloom event, *C. braarudii*’s own co-isolated consortium, and known coccolithophore pathogens tested with a range of outcomes from slightly elevating algal growth to full culture collapse. Close physical association of *P. inhibens* to decalcified *C. braarudii* is likely required to induce cell death, since neither bacterial supernatant nor exposure to growth-inhibiting concentrations of IAA had a detrimental effect on decalcified cultures. Furthermore, exposing calcified and decalcified *C. braarudii* cultures to *P. inhibens* and imaging with scanning electron microscopy (SEM), we find visible bacterial attachment on decalcified cells, but not on calcified cells under the tested conditions. Our results provide the first experimental evidence that the coccosphere can provide a barrier to opportunistic bacterial attack.

## Results

Here, we demonstrate that the coccosphere of calcified, diploid *C. braarudii* cells acts as an effective barrier against opportunistic bacterial attack. Calcified *C. braarudii* cultures are unaffected when exposed to the known *G. huxleyi* pathogen *Phaeobacter inhibens*^34–36^ across a range of initial bacterial concentrations. In contrast, artificially decalcified *C. braarudii* cultures exhibit reduced growth at lower bacterial concentrations and completely collapse due to bacteria-induced cell death at high bacterial concentrations. We observed this collapse of decalcified *C. braarudii* cultures uniquely with *P. inhibens* (and to a lesser degree with a *G.* *huxleyi-*associated bacterium, *Vibrio* 6e7) and not with a range of other coccolithophore-associated bacteria. Furthermore, we found growth inhibition could not be triggered by the addition of high concentrations of indole-3-acetic acid (IAA, 1000 µM) as has been seen with *G. huxleyi*^34,36,37^, where it has been suggested that *P. inhibens* switches from a mutualistic relationship to a pathogenic one partially by up-regulating IAA production. Exposure of *C. braarudii* cultures to *P. inhibens* supernatant likewise did not trigger algal collapse, suggesting that stable diffusible exudates alone are unlikely to be the primary triggers of the observed collapse. In addition, scanning electron microscopy revealed substantial bacterial attachment only on decalcified cells exposed to *P. inhibens*, whereas exposed calcified cells showed no visible bacterial attachment under the testing conditions. Together, the IAA, supernatant, and SEM observations suggest that a close physical association between algae and bacteria may contribute to the observed *C. braarudii* collapse. These findings demonstrate that the coccosphere of *C. braarudii* can serve as a protective barrier to prevent opportunistic attack by pathogenic bacteria, expanding the known functions of the coccosphere in coccolithophores and providing a blueprint for similar investigations in other coccolithophore species.

### Calcification protects *Coccolithus braarudii* from *Phaeobacter inhibens*–mediated cell death

**Fig. 1:**
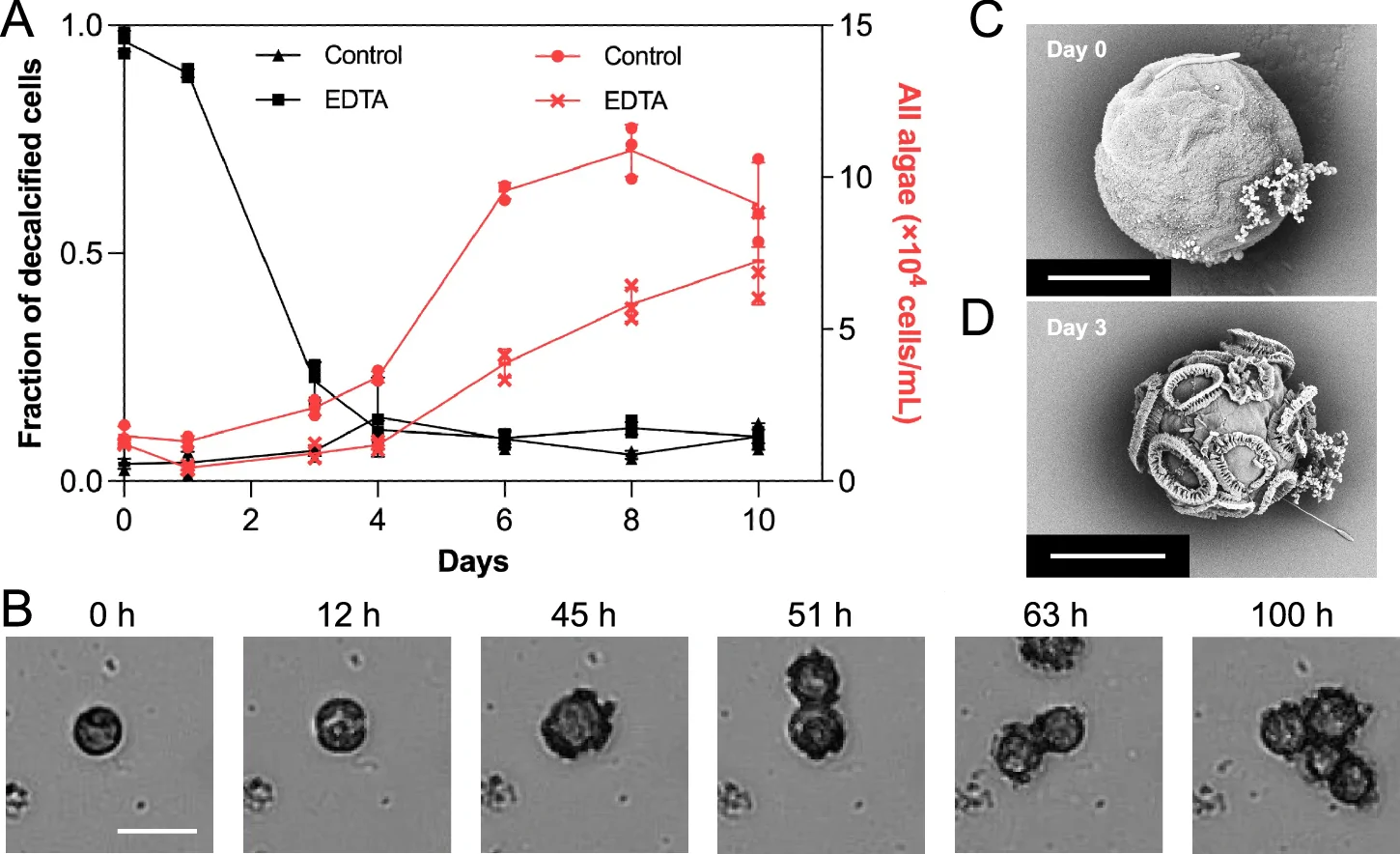
Tracking and imaging of artificially decalcified *C. braarudii* cells. **A** Cell counts (red, right axis) and decalcified fraction (black, left axis) as a function of time for untreated cultures (red circles and black triangles) and cultures decalcified using EDTA (black squares, red crosses). Shows the recalcification and cell growth in the EDTA treatment over the course of ten days. Symbols represent individual biological replicates (*n* = 3), the lines connect mean values, and error bars indicate ±SD. **B** Time-lapse of EDTA-decalcified cells over the course of 100 h, showing recalcification and growth (scale bar = 10 µm). **C**, **D** SEM images of a *C. braarudii* cell (**C**) immediately after decalcification with EDTA and (**D**) 3 days after decalcification (scale bar = 5 µm).

To investigate the role of *C. braarudii’*s coccosphere, we first explored methods to decalcify *C. braarudii* without lasting effects on either the cells’ growth or their ability to recalcify. We found that treatment with ethylenediaminetetraacetic acid (EDTA, 0.02 M EDTA, 5 min, see ‘Materials and methods’) efficiently decalcified the cells. This was confirmed both visually (SI Video S1) and via flow cytometry, with decalcified cells producing reduced forward scatter (FSC-A) and elevated fluorescence signals when compared to their calcified counterparts (SI Fig. S1). We found 95% of the population decalcified with EDTA treatment, compared to only 4% in no-EDTA controls (Fig. 1A). Post-treatment, flow cytometry tracking and time-lapse microscopy revealed that cells began recuperating quickly, with recalcification followed by cell growth progressing in parallel with untreated controls and reaching comparable levels by day 10 (Fig. 1A, B), although with an initial reduction in growth rate in EDTA-treated cultures relative to untreated cultures. Both sets of cultures (treated and untreated) underwent the same washing, spinning, and media replacement steps (see ‘Materials and methods’). Microscopy and flow cytometry confirmed the emergence of coccoliths within three days post-decalcification (Fig. 1B–D) and 90.2% (95% CI: 86.5–94%) of the population recalcified by day 10 post-treatment (Fig. 1A). Similar decalcification and recovery results were also achieved using HCl treatment (1 M HCl for 5 min at pH 2, followed by neutralization to pH 8; SI Fig. S2). We chose to use the EDTA treatment for decalcification in the rest of this work, as it resulted in higher cell growth compared to the HCl treatment (SI Fig. S2) and has been shown to be less likely to damage cellular structures and interfere with physiological processes^38,39^.

The marine bacterium *Phaeobacter inhibens* exhibits a well-characterized ‘Jekyll-and-Hyde’ interaction with the coccolithophore *Gephyrocapsa huxleyi*^34–37^, wherein *P. inhibens* initially promotes algal growth, leading to elevated cell densities relative to unexposed algae cultures. This mutualistic phase is transient, however: upon sensing specific algal-derived signals, *P. inhibens* switches to a pathogenic state, producing bioactive secondary metabolites that induce algal mortality and culture collapse both in calcifying and non-calcifying *G. huxleyi* strains^34,35^. This led us to investigate whether the same interaction occurs between *P. inhibens* and *C. braarudii* cultures along with their co-isolated bacterial community. We combined *C. braarudii*, both calcified and EDTA-decalcified, with different initial concentrations of *P. inhibens* (10^4^ to 10^6^ cells/mL) and monitored the algal population through both light microscopy and flow cytometry. These bacterial concentrations—particularly 10^6^ cells/mL—are on the higher end of what is observed in natural seawater, but fall within the range reported for dense microbial aggregations such as those occurring in phytoplankton blooms, where chemotaxis may further increase localized bacterial concentrations up to 7–200 fold compared to the bulk^40–42^, and bacterial abundances can exceed 10^7^ cells/mL^43,44^. We selected this range to ensure a sufficiently strong and measurable interaction between host and bacterium across treatments, while acknowledging that the upper range likely represents more limited environmental conditions.

**Fig. 2:**
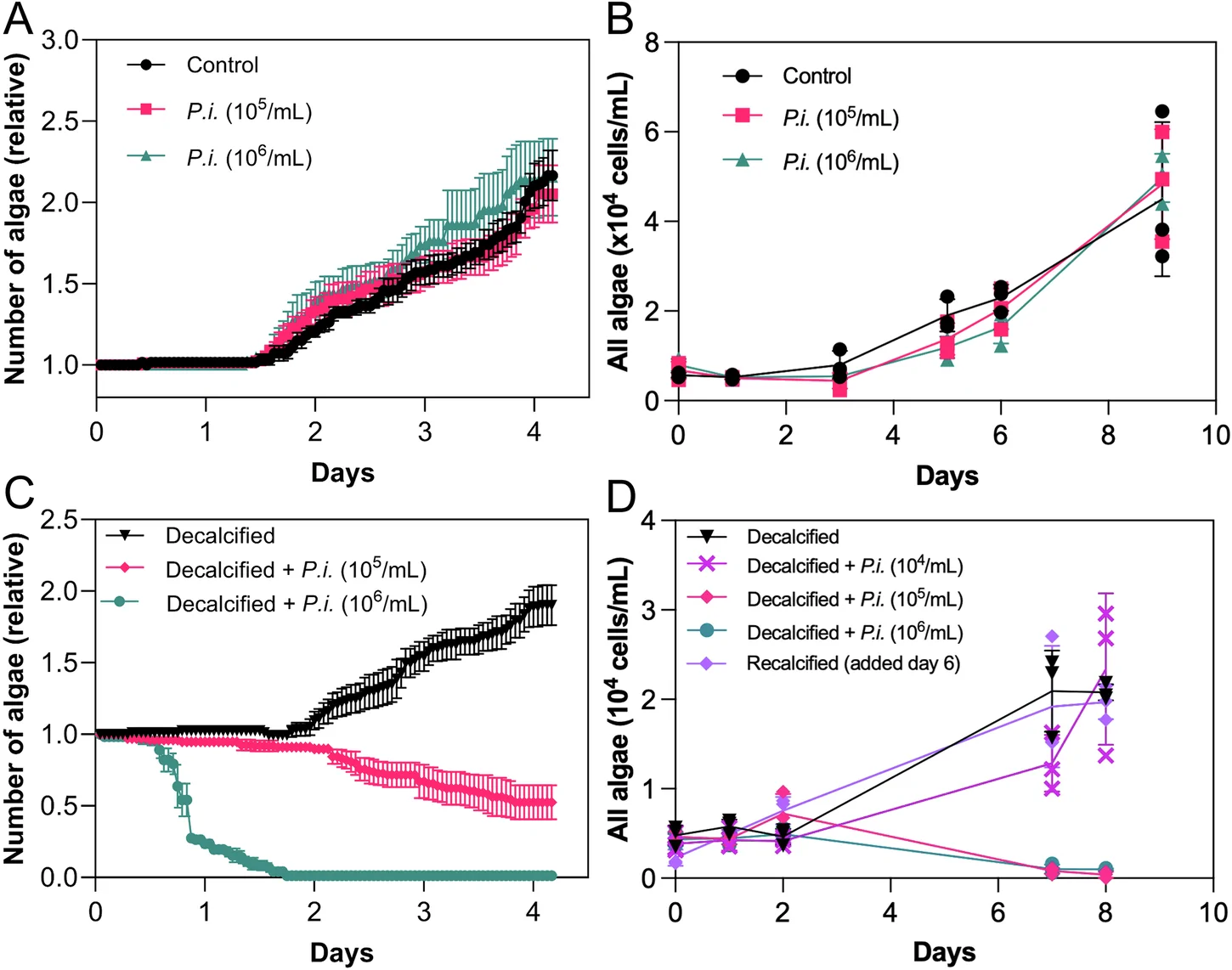
Calcified and decalcified *C. braarudii* show contrasting responses on exposure to *P. inhibens.* Differential response of calcified (**A**, **B**) and EDTA-decalcified (**C**, **D**) *C. braarudii* to *P. inhibens* (‘Pi’) bacteria, assessed via time-lapse microscopy. **A**, **C** and flow cytometry (**B**, **D**). Cell numbers in the microscopy measurements (**A**, **C**) were normalized to those at time 0. Symbols represent the mean of the biological replicates (*n* = 5), the lines connect mean values, and error bars indicate ±SD, all individual replicate data plotted in SI Fig S3. **B**, **D** Symbols represent individual biological replicates (*n* = 3), the lines connect mean values, and error bars indicate ±SD. **A** Single-cell microscopy of calcified *C. braarudii* reveals no significant difference in growth between untreated controls (black circles) and cultures inoculated with *P. inhibens* at 10⁵ (pink squares) or 10⁶ cells/mL (green triangles) (two-way ANOVA, *F*_2,12_ = 0.48, *p* = 0.63; *n* = 5 fields of view). **B** Flow cytometry measurements further confirm no difference in growth between the same three treatments (two-way ANOVA, *F*_2,6_ = 0.24, *p* = 0.80). **C** Single-cell microscopy of EDTA-decalcified *C. braarudii* reveals a marked decrease in cell numbers, compared to the control (black triangles), upon addition of *P. inhibens* at 10⁵ cells/mL (green circles) and 10⁶ cells/mL (two-way ANOVA, *F*₂,₁₂ = 134.4, *p* < 0.0001; Dunnett’s test, *p* < 0.05). The reduction in algal cell number compared to the control was significantly different for all points for 10^6^ from 19 h on and for 10^5^ from 52 h on. **D** Flow cytometry measurements show a dose-dependent decline in decalcified populations exposed to *P. inhibens* at 10⁴ (purple crosses), 10⁵ (pink diamonds), and 10⁶ (green circles) cells/mL. *P. inhibens* was also added on day 6, after the decalcification fraction had dropped to 0.1 (purple diamonds). The reduction in algal cell number compared to the control was significant for 10⁵ and 10⁶ cells/mL on day 7 (10⁶ Pi: *p* = 0.0310; 10⁵ Pi: *p* = 0.0311) and day 8 (10⁶ Pi: *p* = 0.001; 10⁵ Pi: *p* = 0.0003) (two-way ANOVA, *F*₃,₈ = 104.7, *p* < 0.0001; Dunnett’s test).

The growth of calcified *C. braarudii* was unaffected by exposure to *P. inhibens* at either 10^5^ or 10^6^ cells/mL over a 10-day period, as confirmed with both time-lapse microscopy and flow cytometry (Fig. 2A, B, individual microscopy replicate data shown in SI Fig. S3A). In stark contrast, EDTA-decalcified *C. braarudii*, monitored by microscopy, showed marked sensitivity to *P. inhibens* exposure: at 10^5^ cells/mL, cell numbers began to decline after 2 days, while exposure to 10^6^ cells/mL resulted in rapid population collapse within 15 h(Fig. 2C, individual replicate data shown in SI Fig. S3B). Flow cytometry further confirmed this dose-dependent response, also revealing a significant reduction in algal cell numbers for the 10^5^ and 10^6^ treatments but only by days 7–8 (Fig. 2D). In comparison, cultures exposed to 10^4^ *P. inhibens* cells/mL showed no significant difference from untreated controls over the eight days of observation, with only a slight reduction in growth seen at some intermediate time points. Furthermore, when decalcified *C. braarudii* was allowed to fully recalcify, and *P. inhibens* was added six days after the EDTA treatment, there was also no reduction in growth seen over the eight days of flow cytometry observation (Fig. 2D). This lack of response was also observed with partial recalcification (40–60%) of the *C. braarudii* population (SI Fig. S4). These results demonstrate that decalcified *C. braarudii* cells are highly susceptible to *P. inhibens*, but only when nearly fully decalcified and with a sufficiently dense bacterial population. This vulnerability was independent of the decalcification method, with similar results observed using HCl treatment (SI Fig. S2).

Exposure of *C. braarudii* to *P. inhibens* was associated with an increase in the other bacterial populations present in the algal cultures, quantified as colony-forming units (CFUs, SI Fig. S5). Colonies displaying the characteristic brown pigmentation associated with *P. inhibens*^45^ were recorded as putative *P. inhibens* colonies based on morphology. While colony phenotype alone cannot confirm identity in a xenic background, brown colonies were only observed after the addition of *P. inhibens* and were absent when plating the *C. braarudii* consortium alone. (SI Fig. S6). We found that in both calcified and decalcified treatments, addition of *P. inhibens* resulted in an increase in other bacterial populations (SI Fig. S5A). When inoculated in identical conditions (starting bacterial and algal cell densities), *P. inhibens* exhibited markedly faster growth dynamics when co-cultured with decalcified *C. braarudii* cells compared to calcified *C. braarudii* cells. In the decalcified condition, *P. inhibens* grew by more than 22-fold, reaching a peak of 3.7 × 10^7^ cells/mL (95% CI: 7.2–0.2 × 10^7^ cells/mL). In contrast, growth in the calcified treatment was much more limited, with only a 5.5-fold increase to a maximum of 0.83 × 10^7^ cells/mL (95% CI: 1.4–0.3 × 10^7^ cells/mL)—a reduction of 77% in peak abundance compared to the decalcified treatment.

This drastic increase in bacterial growth in the decalcified treatment is even more notable since decalcified *C. braarudii* exhibit slightly reduced growth relative to their calcified counterparts (Fig. 1A). At the onset of algal collapse, as tracked by flow cytometry (around day 2, Fig. 2D)*, P. inhibens* concentrations reached approximately 3.7 × 10^6^ cells/mL while algal concentration was 4.9 × 10^3^ cells/mL (95% CI: 3.4–6.4 × 10^3^ cells/mL), corresponding to approximately 755 bacteria per alga. This represents more than a threefold increase compared to the initial inoculation ratio of 233 bacteria per alga (10^6^ bacterial cells/mL with 0.43 ×  10^4^ algal cells/mL), suggesting that bacterial proliferation relative to the algal population preceded and likely contributed to the observed culture collapse. A subsequent drop in *P. inhibens* abundance occurred in the decalcified treatment after day 7, coinciding with the collapse of the algal population (SI Fig. S5B), suggesting a tight coupling between bacterial and host dynamics in the absence of the coccosphere. This concurrent decrease in concentration likely reflects the exhaustion of algal-derived nutrient sources for *P. inhibens*, suggesting that the pathogenic phase is self-limiting in the absence of a sustained host.

### Strain-specificity and mechanisms underlying the collapse of decalcified *C. braarudii* cultures exposed to *P. inhibens*

**Fig. 3:**
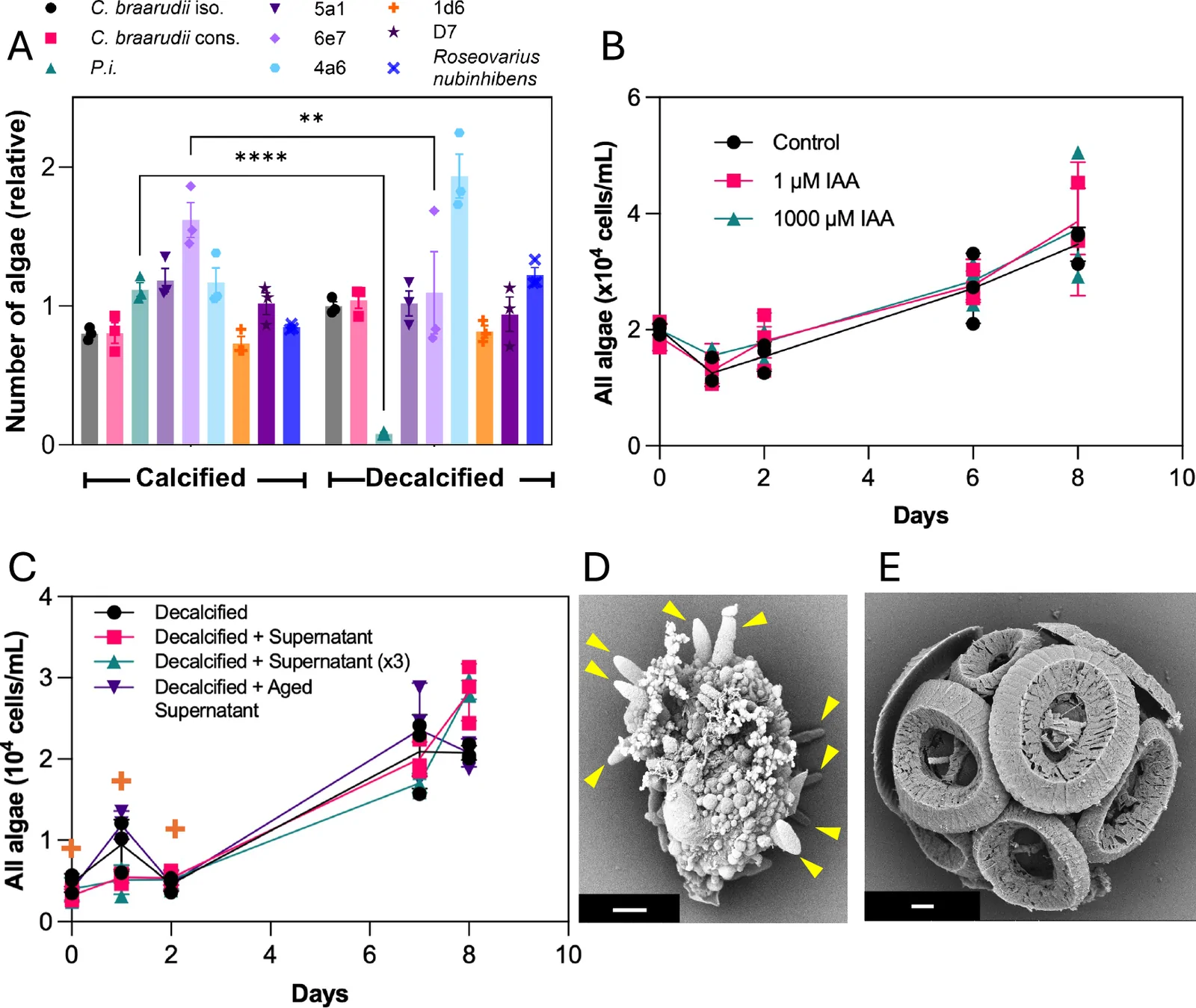
The collapse of decalcified *C. braarudii* cultures upon bacterial exposure is bacterial-strain-specific and is associated with bacterial attachment. **A** Normalized concentrations of calcified and EDTA-decalcified *C. braarudii* after 8 days of exposure to different coccolithophore-associated bacterial strains (different symbols and colors). Cell counts were obtained by flow cytometry and are normalized first to day 0 values and then to the mean cell count of the respective untreated controls (calcified or decalcified) on day 8. Most bacteria had no significant negative impact, except for *P. inhibens* DSMZ17395 and *Vibrio* strain 6e7, which triggered significant decreases in algal cell counts in decalcified but not in calcified cultures, and with 4a6 decalcified treatments grew significantly better than calcified treatments (two-way ANOVA, *F*_8,36_ = 12.69, *p* < 0.0001; Šídák’s post hoc test: DMSZ17395: *p* < 0.0001, 6e7: *p* = 0.01, 4a6: *p* < 0.0001; symbols represent the *n* = 3 independent cultures and the standard error.). **B** Time-resolved cell counts obtained by flow cytometry for decalcified *C. braarudii* exposed to indole-3-acetic acid (IAA) at concentrations previously reported to promote growth (1 µM) or have growth-inhibiting effects (1000 µM) in *G. huxleyi*^34^. No significant effect on cell growth of IAA at either concentration was observed (two-way ANOVA, *F*₂,₆ = 0.95, *p* = 0.44; symbols represent individual biological replicates (*n* = 3), the lines connect mean values, and error bars indicate ±SD). **C** Time-course analysis of decalcified *C. braarudii* populations exposed to various *P. inhibens* cell-free supernatants: (i) a single dose from *P. inhibens* pre-incubated in *C. braarudii* spent cell-free medium, (ii) three doses of the same, delivered on days 0, 1, and 2 (indicated with orange pluses), and (iii) the supernatant from a 7-day-old collapsed co-culture of *P. inhibens* and decalcified *C. braarudii*. None of these treatments led to a significant change in cell numbers (two-way ANOVA, *F*_3,8_ = 0.52, *p* = 0.68; symbols represent individual biological replicates (*n* = 3), the lines connect mean values, and error bars indicate ±SD), suggesting that close physical proximity is likely necessary to induce collapse. **D**, **E** Scanning electron micrographs of decalcified *C. braarudii* cells 3 days post-decalcification. Yellow triangles **D** denote bacteria attached to an EDTA decalcified *C. braarudii* following exposure to 10^6^ cells/mL of *P. inhibens*. **E** No visible attachment was observed on calcified *C. braarudii* cells inoculated with the same concentration of *P. inhibens*. Scale bars = 1 µm.

To determine whether the collapse of decalcified *C. braarudii* cultures is specific to *P. inhibens*, we tested eight additional bacterial strains on artificially decalcified *C. braarudii* cultures and monitored the results using flow cytometry (Fig. 3A). These were: (i) a *C. braarudii*-associated bacterial consortium derived from long-term laboratory cultures, (ii) a single isolate from the long-term laboratory consortium, (iii) four isolates obtained from a *G. huxleyi*-induced mesocosm bloom event in Bergen^40^ (5a1, 6e7, 4a6, 1d6), and (iv) two other known *G. huxleyi* pathogens *Sulfitobacter sp*. D7 and *Roseovarius nubinhibens*^46,47^. Among all treatments, only *P. inhibens* and one further mesocosm isolate, *Vibrio* 6e7, caused a significant decrease in *C. braarudii* cell numbers in decalcified cultures (Fig. 3A). In contrast to *P. inhibens*, the other known *G. huxleyi* pathogens *Sulfitobacter* D7 and *R. nubinhibens* had no effect on *C. braarudii* growth in either calcified or decalcified cultures. Moreover, exposure to both *Alteromonas* 4a6 and the *C. braarudii* consortium resulted in elevated *C. braarudii* cell numbers in decalcified cultures compared to calcified controls. These findings demonstrate that the bacteria-induced collapse of decalcified *C. braarudii* is specific, with different bacterial isolates inducing varying effects on the algal cultures.

To probe at the potential mechanism underlying the collapse of decalcified *C. braarudii* cultures when exposed to *P. inhibens*, we first tested whether the same dose-dependent IAA phenomena present in *G. huxleyi–P. inhibens* system were also responsible for the cell death of *C. braarudii*. With *G. huxleyi–P. inhibens* systems, the transition of *P. inhibens* to a pathogenic state is accompanied by increased production of indole-3-acetic acid (IAA), a growth hormone that—at elevated concentrations—prevents algal growth without direct cell-cell contact or attachment^34–36^. Here, we inoculated calcified and decalcified *C. braarudii* cultures with IAA at two concentrations, 1 and 1000 µM, previously shown to be respectively growth-promoting and growth-inhibiting to *G. huxleyi*^34,36,37^. No significant effect on *C. braarudii* population growth was observed at either concentration with either decalcified (Fig. 3B) or calcified (SI Fig. S7) cultures. These findings indicate that IAA-mediated growth inhibition is not contributing to the observed collapse in decalcified *C. braarudii*. Instead, the interaction is likely driven by other bacterial metabolites and/or requires close physical association with the algae—highlighting a distinct, potentially contact-dependent mechanism.

To further investigate whether close physical association might be necessary for *P. inhibens* to induce the observed cell death in decalcified *C. braarudii*, we tested whether bacterial exudates alone trigger the culture collapse. We designed three treatments utilizing the supernatants of different *P. inhibens* cultures: (i) Single supernatant addition: *P. inhibens* was grown overnight, washed, and then incubated for 24 h in cell-free spent medium from a *C. braarudii* culture. After this incubation, bacterial cells were removed by filtration, and the resulting supernatant was added once (day 0) to a fresh decalcified *C. braarudii* culture. (ii) Repeated supernatant additions: The same supernatant as in point (i) was added on three consecutive days (days 0–2, indicated with green pluses) to test whether cumulative exposure or higher metabolite concentration would induce algal cell death. (iii) Post-collapse co-culture supernatant: Supernatant was collected from a 7-day-old co-culture of decalcified *C. braarudii* and *P. inhibens* that had already induced algal collapse. After removing all cells, this mixture of metabolites was added to a fresh decalcified *C. braarudii* culture to test whether toxic compounds accumulated during collapse could trigger algal cell death without direct bacterial contact. Across all three treatments, no significant changes in algal cell growth were observed over an 8-day period compared to untreated decalcified controls, as measured by flow cytometry (Fig. 3C). Although the involvement of vesicles or labile compounds cannot be excluded from these results, they do indicate that stable diffusible exudates from *P, inhibens* are insufficient to induce cell death in *C. braarudii* and points towards the likelihood that close physical association is required.

The need for close association is further suggested by scanning electron microscopy imaging of the co-cultures, which showed substantial bacterial attachment to decalcified *C. braarudii* treated with *P. inhibens* (Fig. 3D), whereas no visible attachment was observed on calcified cells under the testing conditions. No visible attachment was observed on decalcified cells not treated with *P. inhibens* either (Figs. 3E and 1C), indicating that the attached bacteria in the *P. inhibens*-treated cultures are likely *P. inhibens*, as opposed to members of the natural consortium of *C. braarudii* (however, it is also possible that the presence of *P. inhibens* triggers the attachment of other members of the xenic culture). Together, these findings suggest that the pathogenic behavior of *P. inhibens* could be contact-dependent and support the hypothesis that calcification may shield some phytoplankton from potentially harmful bacterial interactions.

## Discussion

Here, we provide the first direct experimental evidence that the coccosphere of a heavily calcifying species, *C. braarudii*, offers physical protection against bacterial-induced killing. This work was motivated by the long-standing hypothesis that proposes that the tightly interlocked coccoliths of coccolithophores could provide a mechanical barrier against bacterial attack^9^ by reducing or preventing bacterial attachment and penetration. Previous observations of the attachment of *P. inhibens* only to decalcified *G. huxleyi*^34^ suggested the potential connection between the coccosphere and protection against bacteria, which we have now shown systematically on both the single-cell level and in bulk.

Naturally calcified *C. braarudii* cultures (strain RCC1200) were decalcified with brief exposure to EDTA (see ‘Materials and methods’). Control calcified cultures underwent identical handling steps (timing, washing, and media replacements) to the EDTA decalcified treatments, with decalcified cultures slowly regaining their coccosphere over time (~2–3 days, Fig. 1) and regaining final cell counts comparable to their calcified counterparts (Fig. 1A). Upon artificial decalcification, *C. braarudii* cells become susceptible to bacterial attack from specific bacterial strains, including the well-characterized *G. huxleyi* ‘Dr. Jekyll-Mr. Hyde’ pathogen *P. inhibens*. Inoculating artificially decalcified *C. braarudii* cultures with concentrations of *P. inhibens* above 10^5^ cells/mL triggers the onset of algal culture collapse within as little as 15 h (Fig. 2C, D). In contrast, no measurable effect was observed in calcified cultures (Fig. 2A, B) despite a naturally occurring decalcified population fraction of approximately 3.8% (95% CI: 1.1–6.4%, Fig. 1A). As the algae slowly recalcify, they regain their defense against this harmful bacterial interaction: inoculating both partially and fully re-calcified cultures of *C. braarudii* with *P. inhibens* does not induce any difference in population counts compared to the control calcified cultures (Fig. 2D, SI Fig. S4), demonstrating that even partial recalcification protects against pathogenic bacterial behavior.

This interaction appears to have high species-specificity: when tested against six coccolithophore-associated bacteria isolated from an induced mesocosm bloom^40^, only one further strain (*Vibrio* 6e7) negatively impacted the artificially decalcified algae (Fig. 3A). Curiously, one strain, *Alteromonas* 4a6 (Fig. 3A), when added to decalcified and calcified *C. braarudii*, triggered enhanced algal growth in the decalcified treatment compared to the calcified treatment. Although EDTA treatment does have a temporally-limited effect on algal growth (Fig. 1A), the pronounced susceptibility of artificially decalcified *C. braarudii* cannot be explained by this effect alone. Both untreated and EDTA-treated cultures underwent identical handling steps (timing, centrifugation, washing, and media replacements), and treated cultures do re-calcify and continue to grow, albeit with a reduced initial growth rate, and regain their defense to the bacterial interaction as they recalcify (Fig. 2D, SI Fig. S4). Whilst it is not possible to fully account for any potential stress or susceptibility induced by the EDTA treatment, algae also face multiple stressors concurrently in natural environments, for example, bacterial pressure in addition to acidifying conditions, and thus our results could also indicate that opportunistic bacterial attack on algae may become more likely as additional environmental pressures, such as those due to climate change, become more prevalent^6,13,18,23,48,49^.

Our results support the interpretation of the coccosphere functionality for the heavily calcified *C. braarudii* as a defensive ‘shell’ protecting the cell body from opportunistic bacterial interactions and subsequent induced cell death. We show that *P. inhibens* proliferated substantially more in decalcified cultures than in calcified ones (peak bacterial populations of 3.7 × 10^7^ cells/mL and 8.3 × 10^6^ cells/mL, respectively), and in the decalcified algal cultures, the *P. inhibens* population sharply declined after day 7, concurrent with the collapse of the algae culture (SI Fig. S5B). SEM imaging showed extensive bacterial attachment to decalcified cells but no visible attachment to calcified ones under the testing conditions (Fig. 3D, E), demonstrating that the coccosphere of *C. braarudii* could actively prevent bacterial association. It is important to note that because *C. braarudii* cultures remain stable only under xenic conditions, we cannot conclusively determine whether the attached bacteria observed are *P. inhibens* or whether the addition of *P. inhibens* triggered pathogenic or algicidal behavior in one or more members of the existing bacterial community. Nonetheless, whether the response arises from a single species or a community-level interaction, we consider this outcome ecologically relevant, as *C. braarudii* naturally exists within a complex, multi-member bacterial consortium.

The observed bacterial-induced culture crash of decalcified *C. braarudii* is likely to have a chemical component—for example, through the production of algicidal compounds, as seen in *P. inhibens–G. huxleyi* system^34,35^. Despite providing significant coverage of the algal cell (Fig. 3D), the coccosphere is not free of gaps and therefore permeable to small molecules. Thus, diffusible algicidal compounds could reach the cell regardless of the calcification state: this process would be favored by bacteria establishing close contact with the algae. However, we observed no negative effect of *P. inhibens* on calcified *C. braarudii* cultures, suggesting that mere proximity to the cell itself is insufficient and that more direct physical association may be required—perhaps to enable close-range delivery of algicidal compounds or even direct injection into the host cell. This hypothesis is supported by our exudate experiments: exposing decalcified *C. braarudii* cultures to exudates from *P. inhibens* did not induce significant cell death (Fig. 3C), nor did treatment with the phytohormone indole-3-acetic acid (IAA; Fig. 3B, SI Fig. S7)—a compound produced by *P. inhibens* and shown to be growth-inhibiting to *G. huxleyi* at high concentrations (1000 µM) without requiring direct contact^34,36,37^. The lack of any detectable effect from either the exudate treatments or high IAA concentrations supports the idea that killing in the *P. inhibens– C. braarudii* system is not solely mediated by diffusible compounds but requires close physical contact normally prevented by the coccosphere. Whilst the algicidal compounds responsible may have short half-lives or may be vesicle-associated, which would likely not be preserved in our static exudates, their transient nature would necessitate close physical association between the algal and bacterial cells. Future work should seek to identify the specific compound(s) responsible for the algal cell death. These could be markedly different from those implicated in bacterial-induced mortality in *G. huxleyi*^34–37^, which succumbs to *P. inhibens* regardless of calcification state. The *P. inhibens–C. braarudii* system highlights a functionally distinct alga-bacterium pairing, and offers a promising model for disentangling species-specific microbial pathogenesis and the evolutionary diversification of algal defenses.

Whether the mortality observed reflects the action of *P. inhibens* alone or a broader community response, the protective role of the coccosphere likely operates through direct physical mechanisms that limit bacterial access to the cell surface. Supporting this hypothesis, previous genomic studies have shown that *P. inhibens* encodes structural genes for a Type VI secretion system (T6SS), which in other species (e.g., *Campylobacter jejuni*) delivers toxic effectors directly into target cells through physical contact using a tube-like structure^50,51^, and can be impaired by extracellular barriers such as capsules or matrices^52^. While T6SS functionality in *P. inhibens* DSM 17395 has not been experimentally shown, if active, it could explain why the pathogenic effect on naked cells can be neutralized by the presence of the coccosphere. Furthermore, the individual coccoliths forming the coccosphere are coated with coccolith-associated polysaccharides (CAPs), which form a dense, negatively charged barrier^53,54^ that could also disrupt molecular cues required for bacterial attachment. *P. inhibens* forms polar N-acetylglucosamine-rich adhesins to establish surface contact and form rosettes^55,56^: interference with these structures could prevent direct attachment required for pathogenesis.

For the heavily calcifying coccolithophore *C. braarudii*, our findings demonstrate that at least part of the coccosphere functionality is as a barrier to protect the cells against opportunistic bacterial attack. This adds to growing evidence that structural armoring in phytoplankton may have appeared, in part, as a defense against biotic threats—not only grazers but also microbial pathogens—mirroring trends observed in other lineages of mineralized or spiny phytoplankton^57–59^. We propose that this is achieved by forming a biochemically selective interface that disrupts both bacterial attachment and contact-mediated effector delivery. This dual function highlights the broader ecological role of calcification in modulating host–microbe interactions. Environmental stressors that compromise coccosphere integrity could significantly increase coccolithophore susceptibility to bacterial attack, altering bloom dynamics and disrupting the intricate balance between algae, bacteria, and viruses—interactions that ultimately drive large-scale ecological events and nutrient fluxes across marine ecosystems.

## Materials and methods

### Algal strains and growth conditions

The xenic diploid strain *Coccolithus braarudii* (RCC1200, obtained from the Roscoff Culture Collection) was grown at 14 °C on a 12:12 diurnal cycle at light intensity of 30 µmol m^−2^ s^−1^ in L1 supplemented synthetic ocean water (SOW, pH = 8)^60^.

### Artificially removing the coccosphere

For EDTA decalcification, 0.02 M EDTA was added to the algal culture for five min^61^, then the cultures were washed and resuspended (1200 relative centrifugal force, RCF, for 5 min) in fresh L1 SOW media. Control (calcified) samples followed identical washing and resuspension treatments. Cell counts for the decalcified treatments were then adjusted to be commensurate with the washed calcified treatments using flow cytometry (see ‘Methods’ Cell Counting).

For HCl decalcification, 1 M HCl was added drop-wise until the culture medium reached pH 2. Cultures were briefly agitated by manual shaking and then neutralized back to pH 8.2 with 1 M NaOH^10^. The decalcified and calcified controls were then washed and resuspended (1200 RCF, 5 min) in fresh L1 SOW media. Cell counts for control and treatment conditions were adjusted to the same starting cell densities using flow cytometry (see ‘Methods’ Cell Counting).

### Bacterial growth and addition to algal cultures

Bacterial strains *Marinomonas* 5a1, *Vibrio* 6e7, *Alteromonas* 4a6, and 1d6 were previously isolated from an induced *G. huxleyi* mesocosm bloom in Bergen^40^, DMSZ17395 (*Phaeobacter inhibens*) was obtained from the Leibniz Institute DSMZ-German Collection of Microorganisms, and *Roseovarius nubinhibens* and *Sulfitobacter sp*. D7 were isolated and provided by Assaf Vardi. The *C. braarudii* RCC1200 bacteria isolate and consortium were isolated from the RCC1200 algal culture using 100% Marine Broth medium (Difco, CAS 10043-52-4) agar plates. All strains were preserved in 20% glycerol solution and stored at −80 °C. Bacteria were inoculated in 100% Marine Broth from frozen stocks 24 h prior to use and grown overnight at 30 °C with constant agitation (300 revolutions per minute, RPM). Cultures were collected at the stationary phase at optical density (OD) 0.3–0.4 (SI Fig. S8) and were washed and resuspended three times (4000 RCF, 5 min) in SOW. The bacteria were later counted using SYBR Green staining and flow cytometry (see ‘Methods’ Cell Counting). 2 mL aliquots of *C. braarudii* cultures were inoculated at the stationary phase (14 days old) with the washed bacteria at the specified concentrations (see below ‘Cell counting’) in sterile, clear 24-wellplates (Falcon) and were then incubated at 14 °C on a 12:12 diurnal cycle at a light intensity of 30 µmol m^−2^ s^−1^.

For supernatant experiments (Fig. 3C), a co-culture was prepared from *P. inhibens*, which was grown and washed as specified above; however, in the final washing step, the medium was replaced with either *C. braarudii* culture or cell-free *C. braarudii* supernatant rather than SOW. These *P. inhibens* cultures were then incubated for 24 h at 14 °C on a 12:12 diurnal cycle at a light intensity of 30 µmol m^−2^ s^−1^ before cells were removed (0.2 µm filter, Filtropur) and the resultant filtrate used to inoculate separate 2 mL aliquots of *C. braarudii* culture with the filtered spent medium at a 1:10 filtrate:algae culture volume ratio in sterile, clear 24-wellplates (Falcon).

For experiments testing the effect of indole-3-acetic acid (IAA, Fig. 3B, Fig. S7), IAA was prepared fresh before each experiment in L1 SOW medium with filter sterilization (0.2 µm filter, Filtropur). IAA was first dissolved in 50% ethanol prepared with L1 medium to ensure solubility, then diluted into the algal culture to a final ethanol concentration of ≤0.1%. This low concentration is well below the 1% threshold previously shown to have no effect on algal growth^37^.

### Microscopy and cell counting

*C. braarudii* cells in four different treatment conditions (either calcified or decalcified, and with/without addition of *P. inhibens*) were gently pipetted into a six-channel IBIDI chip (200 μL per channel, μ-Slide uncoated VI 0.4, Cat. No. 80601) and the ports sealed with strips of parafilm. The chip was imaged using phase-contrast microscopy (Nikon Ti-E; 10× 0.3 NA objective), with an image of the same area taken once per hour for a period of 100 h using a CMOS camera (Orca-Flash 4.0 Hamamatsu), and cells counted manually. The chip was incubated under the microscope using an incubator, and a low-angle ring light (LA-120W, Smart View) was placed around the chip and connected to a digital timer (Brennenstuhl). This allowed for the maintenance of the same conditions in the Algaetron: 14 °C with the ring light set to 30 μmol m^−2^ s^−1^ using a light meter (WALZ ULM 500) with a spherical sensor.

Flow cytometry was used to count both algal and bacterial cells using a cytometer (Beckman Colter, CytoFLEX S) equipped with a 488 nm laser. For algal cell counts, forward scatter (FSC) and red autofluorescence (filter for Peridinin–chlorophyll protein complex PerCP-A) were recorded. For bacterial counts, separate aliquots were stained with SYBR Green (5 µM final concentration, Sigma Aldrich CAS 163795-75-3) and incubated for 10 min in the dark. Cell counts were then obtained by recording FSC and green fluorescence (filter for fluorescein, FITC).

Bacterial counts were additionally obtained as counts of colony-forming units (CFUs) after plating. Aliquots of 100 µL sequentially diluted culture were spread onto 100% Marine Broth Agar (Difco, CAS 90000-518) and incubated at 30 °C for 48 h before counting. Among samples at different dilutions, plates with ~100 colonies were counted. In quantifying CFUs, putative *P. inhibens* colonies were distinguishable from other bacteria in the culture due to their characteristic formation of brown colonies^45^ after 48 h (SI Fig. S5).

### Scanning electron microscopy

In total, 200 µL samples of *C. braarudii* liquid cultures were fixed with 1% (w/v) glutaraldehyde for 1 h, then gently pipetted onto 0.01% poly-L-lysine coated hydrophilized silicon wafers. The wafers were then incubated at ambient temperature for 10 min, then sequentially immersed in 2.5% glutaraldehyde solution (SOW, 27 practical salinity units, PSU), 1% osmium tetroxide, and SOW, for 5 min each. Next, the wafers underwent an ethanol drying series consisting of 2 min immersion in 0, 30, 50, 70, 90, and 100% ethanol, followed by three times water-free 100% ethanol. Critical point dryer (CPD 931 Tousimis, ETH Zurich Microscope Facility ScopeM) with a cell monolayer protocol was then used, and the wafers adhered to aluminum stubs with silver paint. After 24 h degassing, the wafers were sputter-coated with 4 nm platinum palladium (CCU-010 Metal Sputter Coater Safematic, ETH Zurich Microscope Facility ScopeM). Cells were imaged using an extreme high resolution (XHS) TFS Magellan 400 microscope (50 pA current, 5 kV accelerating voltage, ETH Zurich Microscope Facility ScopeM). Backscattered electrons were imaged with a CBS detector and secondary electrons with a TLD detector, both using immersion.

### Statistics and reproducibility

Graphpad Prism 10.4.1 (GraphPad Software, La Jolla, CA, USA) was used for all statistical analyses and graphing. In all cases, a *p*-value of *p* < 0.05 was considered statistically significant. Symbols represent individual biological replicates, the lines connect mean values, and error bars indicate ±SD.

### Reporting summary

Further information on research design is available in the Nature Portfolio Reporting Summary linked to this article.

## Supplementary information

Supplementary Information

Description of Additional Supplementary Materials

Supplementary Video 1

Reporting Summary

## Data Availability

Data files used during the current study are publicly available at the ETH Research Collection, which can be accessed at this 10.3929/ethz-b-000745131^62^.
