# Supplementary material for: The coccosphere of the heavy calcifying coccolithophore Coccolithus braarudii provides defense against bacteria
Source: Commun Biol. 2026 Jul 3;9:1123. doi: 10.1038/s42003-026-10585-2 (PMC13493860; doi:10.1038/s42003-026-10585-2)
Supplement: Supplementary file 1 [file 42003_2026_10585_MOESM1_ESM.pdf]

**SUPPLEMENTARY INFORMATION FOR**

# The coccosphere of the heavy calcifying coccolithophore *Coccolithus braarudii* provides defense against bacteria

Sophie T. Zweifel[1], Richard J. Henshaw[1], Roberto Pioli[1], Clara Martínez-Pérez[1,2], Uria Alcolombri[1,3], Zachary Landry[1,4] and Roman Stocker[1,#]

Author affiliations: [1]Institute of Environmental Engineering, Department of Civil, Environmental and Geomatic Engineering, ETH Zurich, Zurich, Switzerland; [2]Limnological Research Station, Department of Hydrology, University of Bayreuth, Bayreuth, Germany; [3]A. Silberman Institute of Life Sciences, Faculty of Sciences, The Hebrew University of Jerusalem, Jerusalem, Israel; [4]Department of Biological Sciences, University of Southern California, Los Angeles, CA, USA

[#]For correspondence/requests for materials. Email: romanstocker@ethz.ch

Supplementary Figure 1. **Flow cytometry gating for the identification of calcified and decalcified cells in *C. braarudii* population.**

Supplementary Figure 2. **Comparison of decalcification methods for *Coccolithus braarudii***

Supplementary Figure 3. **Individual biological replicate trajectories for calcified and EDTA-decalcified *C. braarudii* exposed to *P. inhibens* monitored with timelapse microscopy.**

Supplementary Figure 4. **Response of EDTA-decalcified *C. braarudii* to *P. inhibens* added at different calcification states assessed by flow cytometry**.

Supplementary Figure 5. **Counts of *Phaeobacter inhibens* and other bacteria when inoculated into calcified and decalcified *Coccolithus braarudii* cultures.**

Supplementary Figure 6. **Comparison of bacteria colonies formed with different *Coccolithus braarudii* treatments.**

Supplementary Figure 7. **Cell growth response of calcified *C. braarudi*i cultures to addition of indole-3-acetic acid.**

Supplementary Figure 8. **Optical density growth curve of *P. inhibens* measured by plate reader.**

Supplementary Video S1. ***Coccolithus braarudii* recalcification and cell division.** Timelapse microscopy video (one frame per hour, total duration 100 h) showing formation of coccoliths and cell division in EDTA decalcified *C. braarudii* cells.

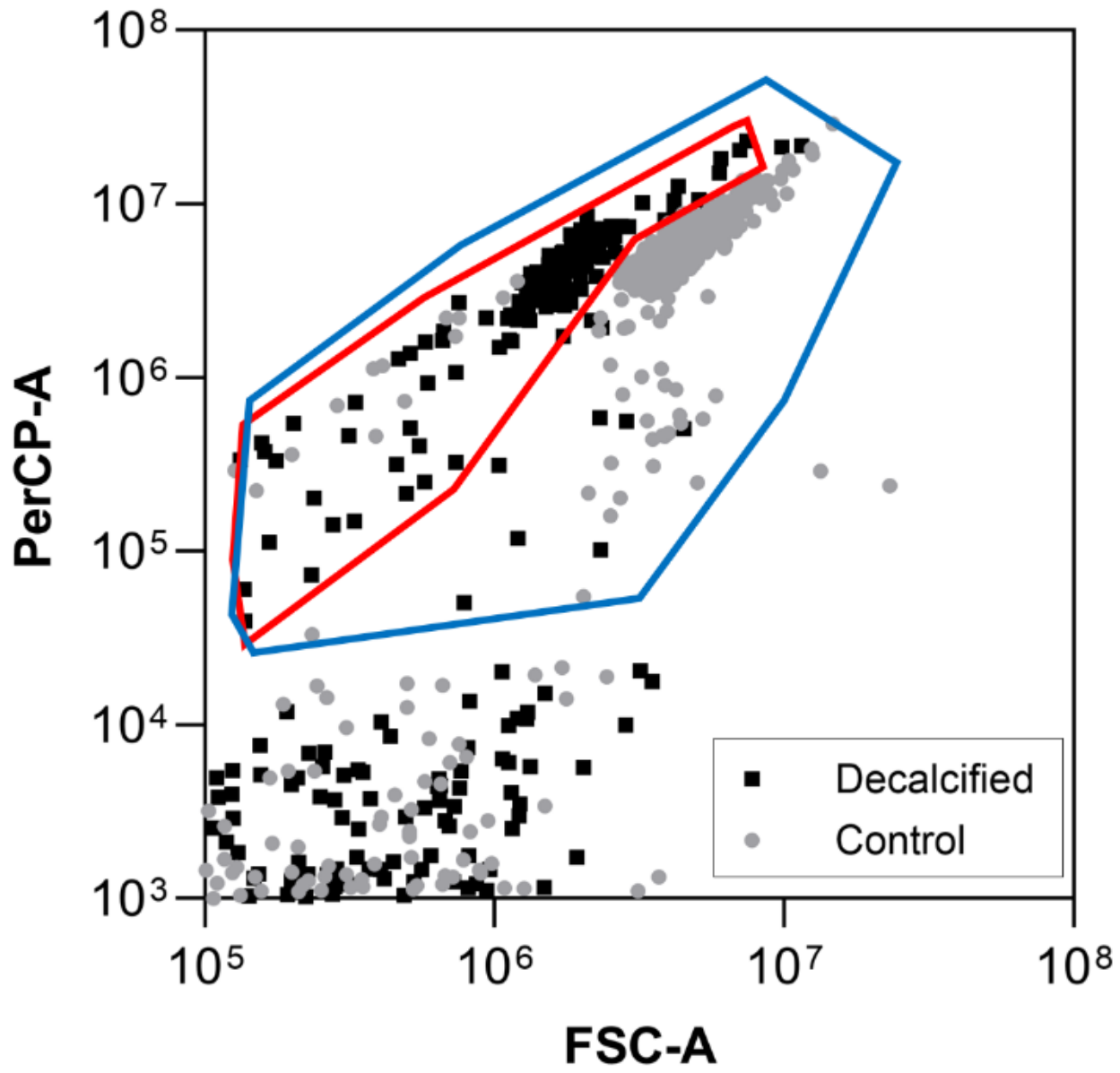


**Supplementary Figure 1. Flow cytometry gating for the identification of calcified and decalcified cells in *C. braarudii* population.** Flow cytometry shows distinct clustering between decalcified (black squares) and control (grey circles) treatments. Populations were identified based on chlorophyll autofluorescence (PerCP-A), with decalcified and control (calcified) cells occupying separate regions of the plot, indicating a shift in cellular optical properties following decalcification with EDTA.

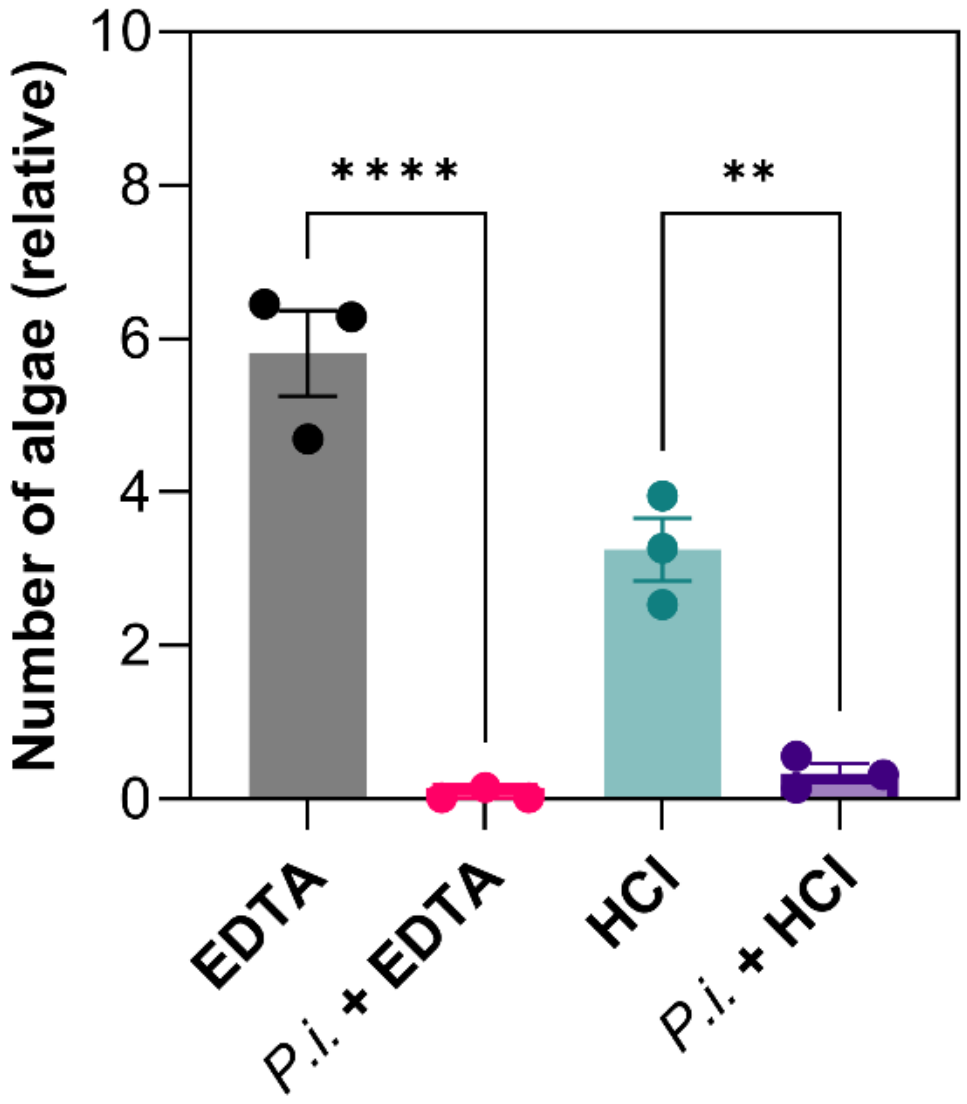


**Supplementary Figure 2. Comparison of decalcification methods in *Coccolithus braarudii* and their effects on susceptibility to *Phaeobacter inhibens*.** *C. braarudii* cultures were decalcified with either HCl (1 M) or EDTA (0.5 M). For each treatment, control conditions received no treatment whereas +*P.i.* (*P. inhibens*) treatments were inoculated with bacteria ($10^6$ cells/mL). Bars show the mean with SE bars for $n = 3$ biological replicates (shown as individual points). Comparison of treatments, two-way ANOVA ($F_{3,6} = 66.78$, $p < 0.0001$). Both *P. inhibens* treatments differ significantly from their respective control (two-tailed Tukey's multiple comparisons test, EDTA $p < 0.0001$, HCl $p = 0.0033$). Algal concentration was normalized to the day zero counts for each treatment group.

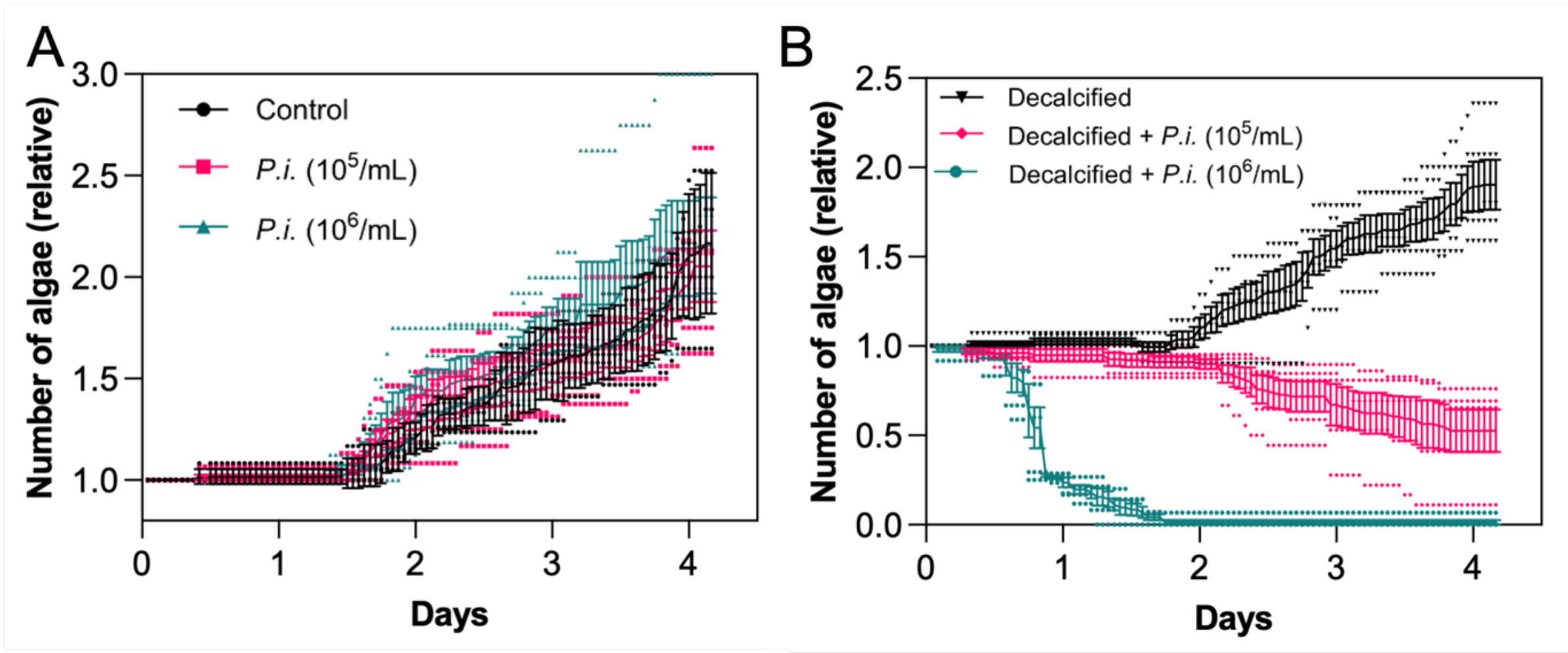


**Supplementary Figure 3. Individual biological replicate trajectories for calcified and EDTA-decalcified C. braarudii exposed to P. inhibens monitored with timelapse microscopy.** Full replicate-level representation of the time-lapse microscopy data summarized in Fig. 2A and Fig. 2C. Cell numbers were normalized to those at time 0. Symbols represent individual biological replicates (*n* = 5) and error bars indicate ± SD. (A) Calcified C. braarudii exposed to P. inhibens at $10^5$ or $10^6$ cells/mL showed no marked reduction in cell numbers compared with the untreated control. (B) EDTA-decalcified C. braarudii exposed to P. inhibens at $10^5$ or $10^6$ cells/mL showed a marked decline in cell numbers compared with the untreated control.

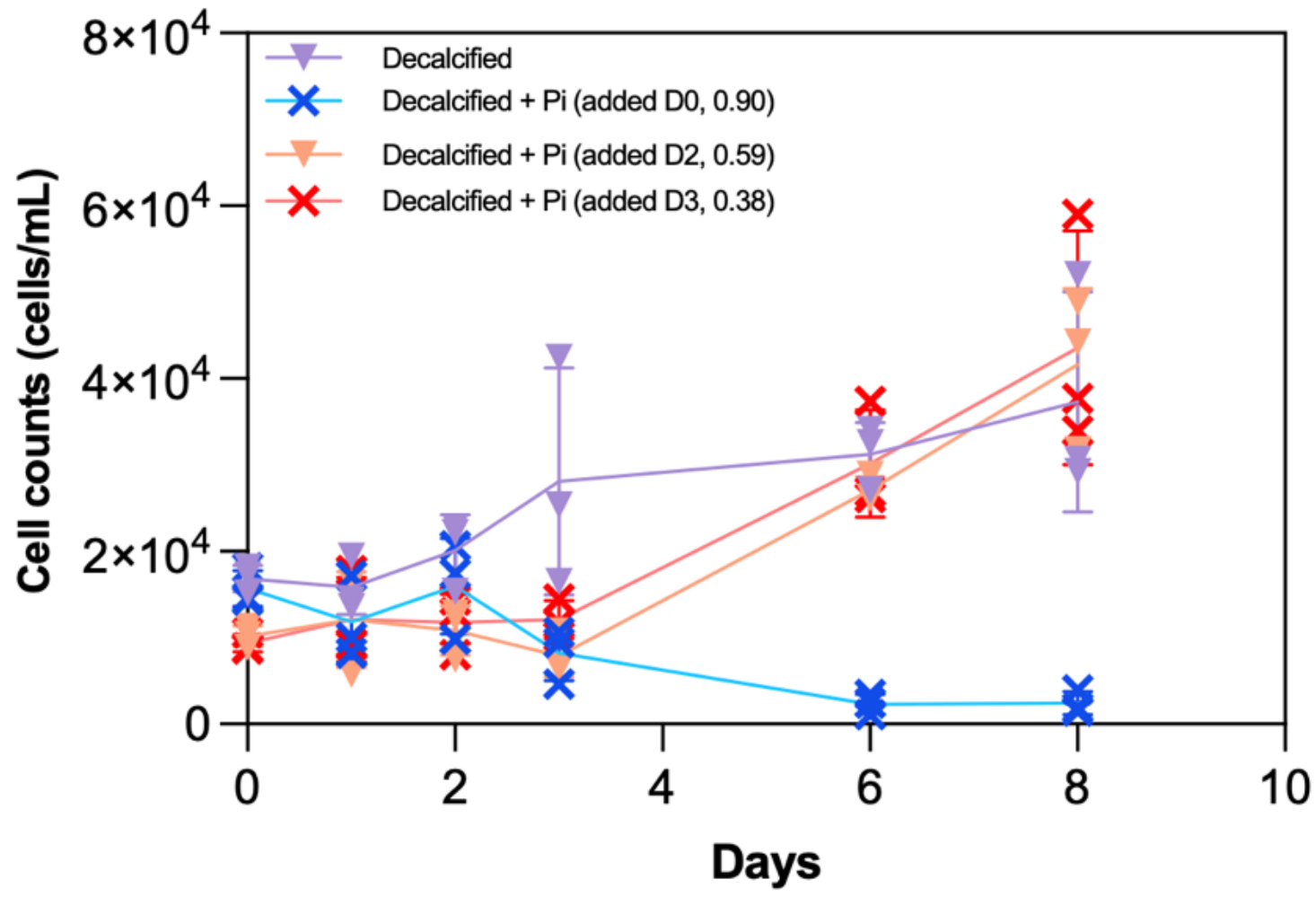


**Supplementary Figure 4. Response of EDTA-decalcified *C. braarudii* to *P. inhibens* added at different calcification states assessed by flow cytometry**. Flow cytometry measurements show that decalcified populations exposed to *P. inhibens* are only susceptible when bacteria are added right after decalcification (blue crosses, 90% decalcified fraction), and not when bacteria are added 48 hours (orange inverted triangles, 60% decalcified fraction) or 72 hours (red crosses, 40% decalcified fraction) later. Symbols represent individual biological replicates ($n$ = 3), the lines connect mean values, and error bars indicate ± SD. The reduction in algal cell number compared to the control was significant for blue crosses (*P. inhibens* added on day 0) on day 6 ($p$ = 0.006) and for inverted orange triangles (*P. inhibens* added at 48 hours) and red crosses (*P. inhibens* added at 72 hours) on day 0 (orange triangles: $p$ = 0.01; red crosses: $p$ = 0.008) (two-way ANOVA, $F_{3,8}$ = 57.0, $p$ < 0.0001; Dunnett's test, n = 3 independent cultures).

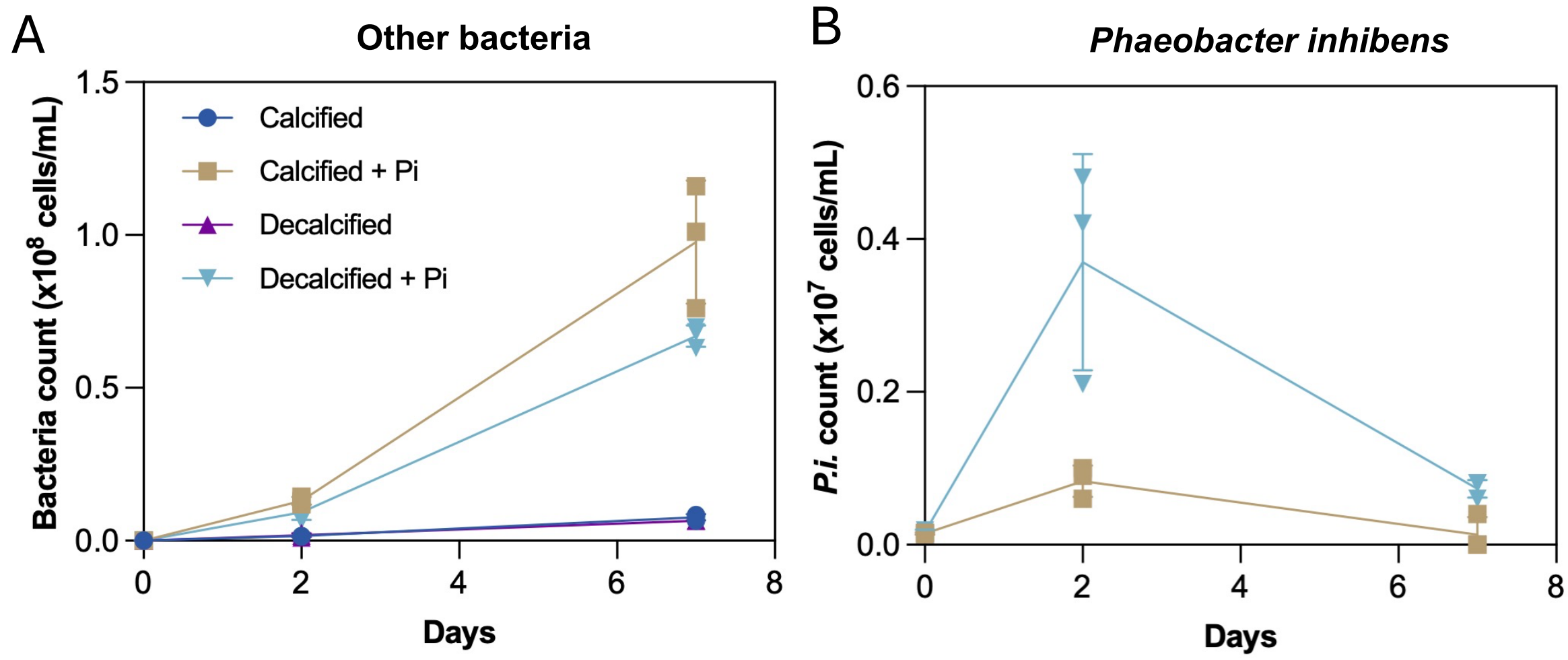


**Supplementary Figure 5. Counts of *Phaeobacter inhibens* and other bacteria when inoculated into calcified and decalcified *Coccolithus braarudii* cultures.** Symbols represent individual biological replicates (*n* = 3), the lines connect mean values, and error bars indicate ± SD. Bacteria counts as a function of time for (A) Punitively identified *P. inhibens* colonies*,* monitored by plating samples on 100% marine broth agar plates and counting colony forming units (CFUs), for calcified *C. braarudii* cultures (blue circles), decalcified cultures (purple triangles), calcified cultures inoculated with *P. inhibens* (brown squares), and decalcified cultures inoculated with *P. inhibens* (inverted light blue triangles). Bacterial growth was significantly different from 2 days on for all points between blue circles and brown squares, and between brown squares and purple triangles, and for 7 days growth was also significantly different between blue circles and inverted light blue triangles and between purple triangles and inverted light blue triangles (two-way ANOVA $F_{3,21} = 56.19$, $p < 0.0001$; Tukey's multiple comparison test: *n* = 3 biological replicates) and (B) all other bacteria in the algal culture. Bacterial growth was not significantly different between brown squares and inverted light blue triangles on any of the three treatments days (two-way ANOVA, $F_{1,10} = 12.28$, $p = 0.0057$; Šídák's multiple comparison test, n = 3 biological replicates).

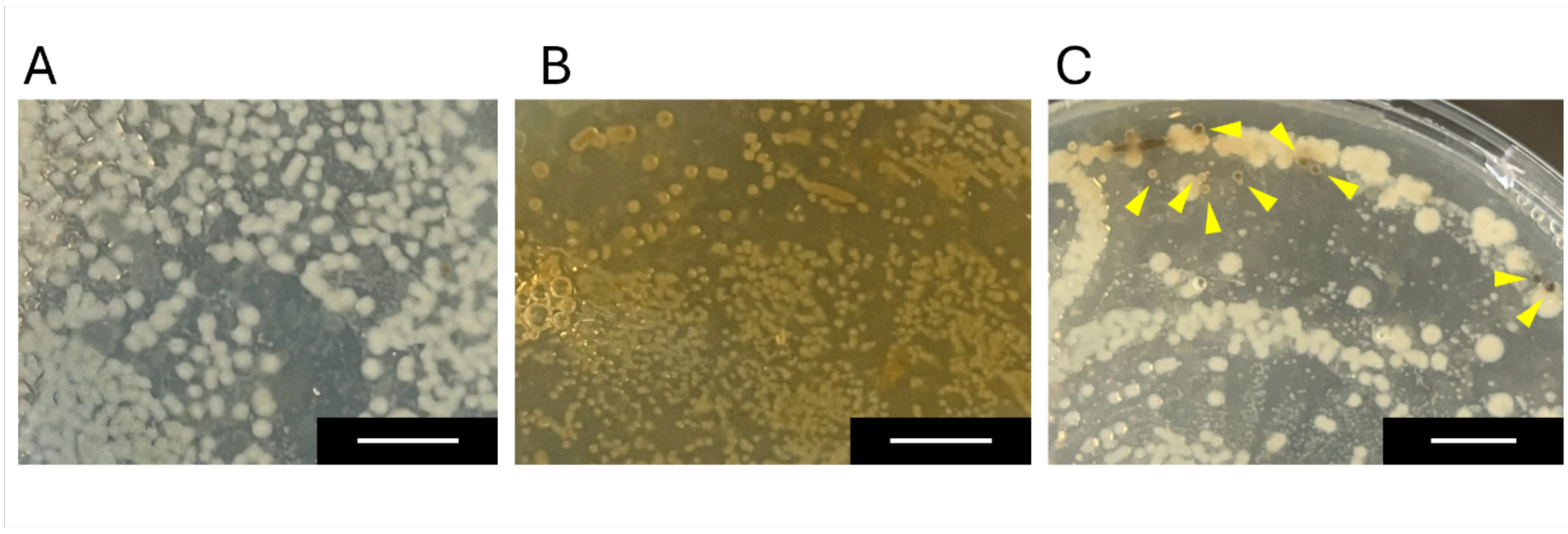


**Supplementary Figure 6. Bacterial colonies formed by communities from different *C. braarudii* treatments.** Colony forming units seen on 100% marine broth agar plates after 48 h incubation at 27 °C for (A) xenic *C. braarudii* culture (B) pure *P. inhibens* culture, (C) xenic *C. braarudii* culture inoculated with *P. inhibens* (*P. inhibens* colonies indicated with yellow triangles). Scale bars = 0.5 cm.

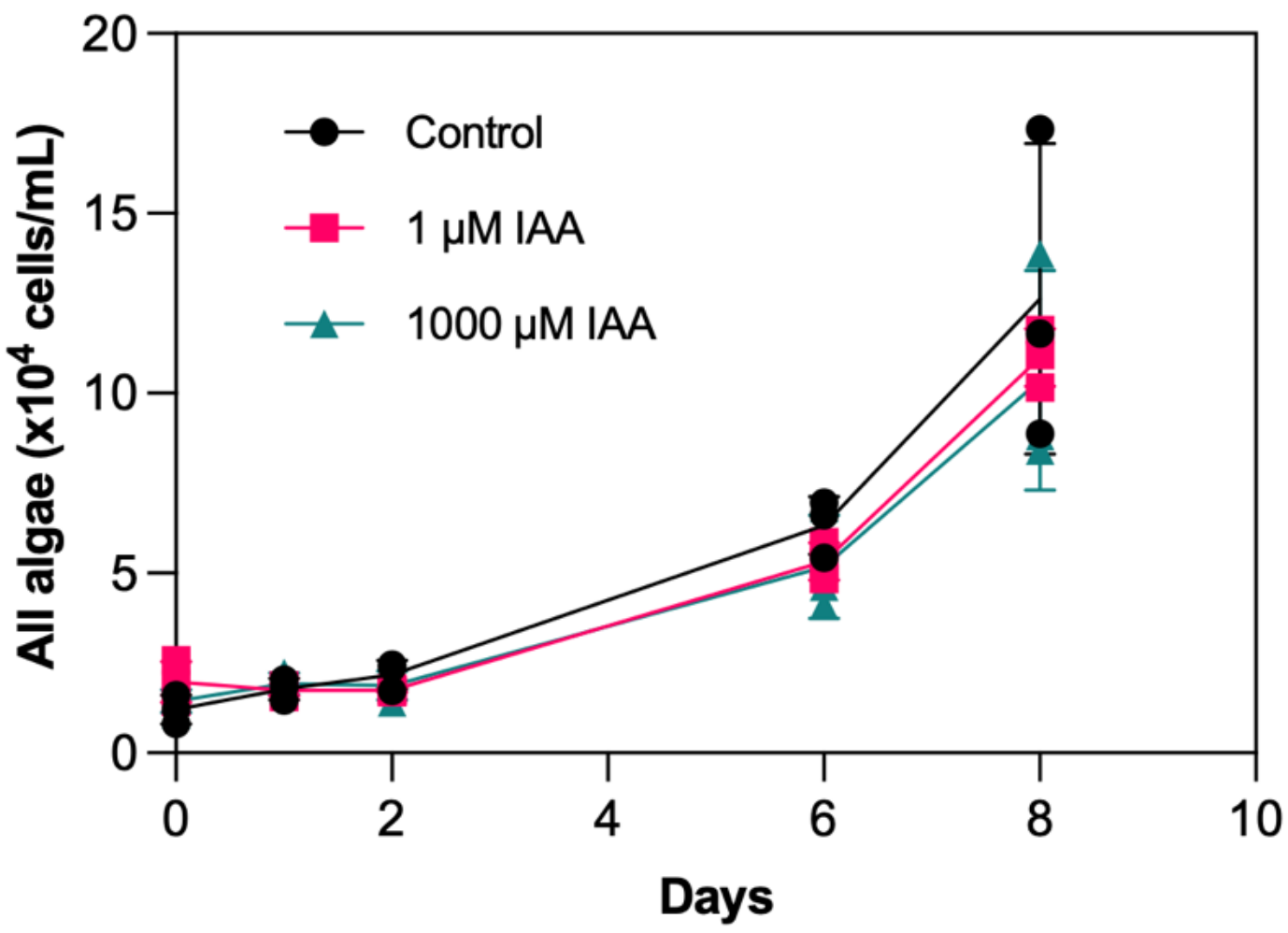


**Supplementary Figure 7. Growth of calcified *C. braarudii* cultures upon addition of indole-3-acetic acid (IAA).** Cell concentrations obtained by flow cytometry as a function of time for an untreated calcified *C. braarudii* culture (black circles) and for calcified cultures treated with indole-3-acetic acid at 1 µM (pink squares) or 1000 µM (green triangles). Symbols represent individual biological replicates ($n$ = 3), the lines connect mean values, and error bars indicate ± SD. Comparison of treatments, two-way ANOVA, $F_{2,6} = 0.81$, $p = 0.49$.

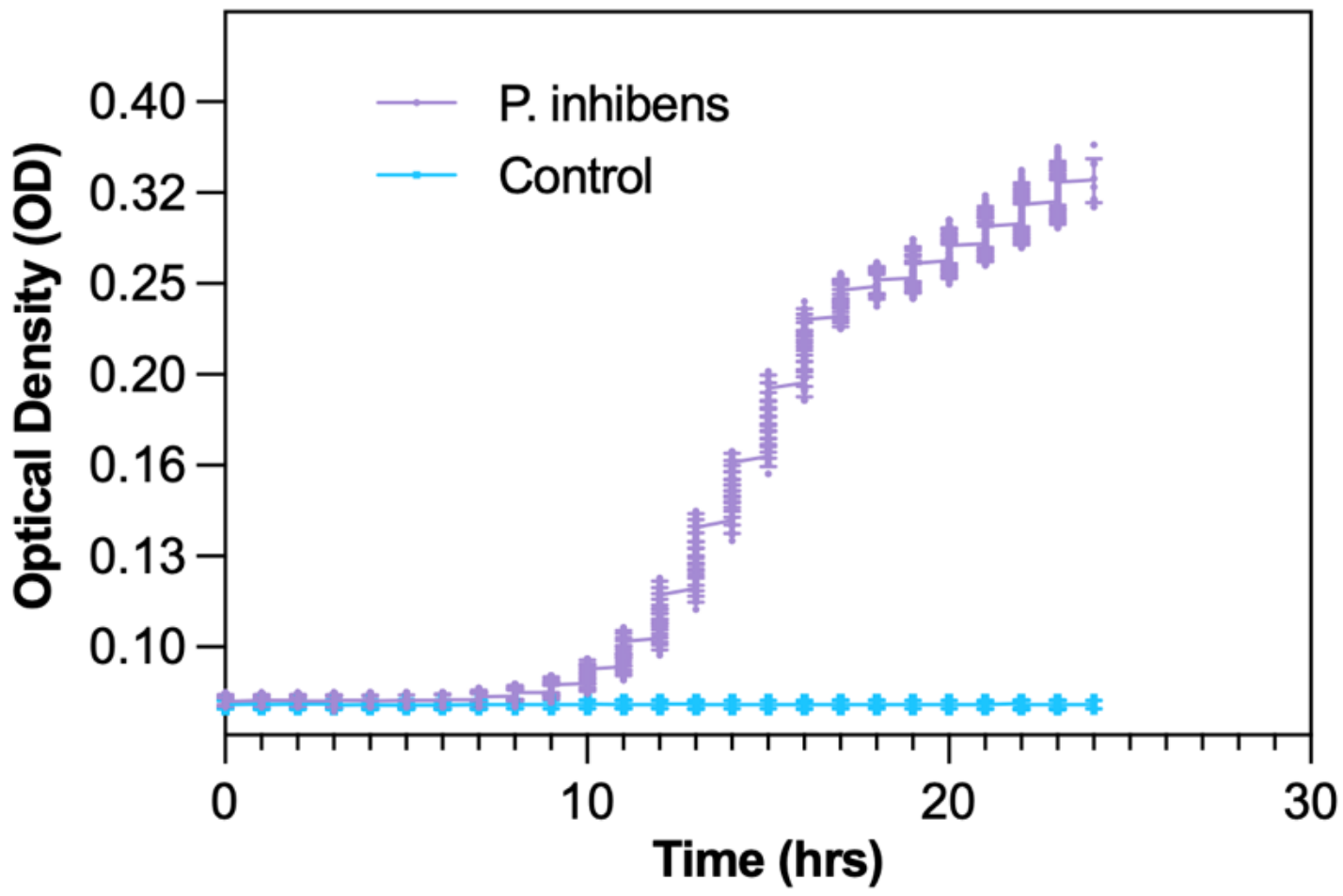


**Supplementary Figure 8. Optical density growth curve of *P. inhibens* measured by plate reader.** Growth of *Phaeobacter inhibens* was monitored by optical density at 600 nm ($OD_{600}$) over 24 h using a 96-well plate. Measurements were collected every 6 min at 30 °C with continuous orbital shaking. Symbols represent individual biological replicates ($n$ = 8), the lines connect mean values, and error bars indicate ± SD. Purple circles indicate wells inoculated with *P. inhibens*, while blue squares represent control wells containing growth medium without bacteria.
