# Supplementary material for: The coccosphere of the heavy calcifying coccolithophore Coccolithus braarudii provides defense against bacteria
Source: Commun Biol. 2026 Jul 3;9:1123. doi: 10.1038/s42003-026-10585-2 (PMC13493860; doi:10.1038/s42003-026-10585-2)
Supplement: Supplementary file 2 [file 42003_2026_10585_MOESM2_ESM.pdf]

Description of Additional Supplementary Files

File name: Supplementary Video 1

Description: *Coccolithus braarudii r*ecalcification and cell division. Timelapse microscopy video (one frame per hour, total duration 100 h) showing formation of coccoliths and cell division in EDTA decalcified *C. braarudii* cells
