# Supplementary material for: The coccosphere of the heavy calcifying coccolithophore Coccolithus braarudii provides defense against bacteria
Source: Commun Biol. 2026 Jul 3;9:1123. doi: 10.1038/s42003-026-10585-2 (PMC13493860; doi:10.1038/s42003-026-10585-2)
Supplement: Supplementary file 3 [file 42003_2026_10585_MOESM4_ESM.pdf]

nature portfolio

Corresponding author(s): Roman Stocker

Last updated by author(s): May 26, 2026

Reporting Summary

Nature Portfolio wishes to improve the reproducibility of the work that we publish. This form provides structure for consistency and transparency in reporting. For further information on Nature Portfolio policies, see our Editorial Policies and the Editorial Policy Checklist.

Statistics

For all statistical analyses, confirm that the following items are present in the figure legend, table legend, main text, or Methods section.

n/a	Confirmed	
☐	☒	The exact sample size (*n*) for each experimental group/condition, given as a discrete number and unit of measurement
☐	☒	A statement on whether measurements were taken from distinct samples or whether the same sample was measured repeatedly
☐	☒	The statistical test(s) used AND whether they are one- or two-sided<br>*Only common tests should be described solely by name; describe more complex techniques in the Methods section.*
☒	☐	A description of all covariates tested
☒	☐	A description of any assumptions or corrections, such as tests of normality and adjustment for multiple comparisons
☐	☒	A full description of the statistical parameters including central tendency (e.g. means) or other basic estimates (e.g. regression coefficient) AND variation (e.g. standard deviation) or associated estimates of uncertainty (e.g. confidence intervals)
☐	☒	For null hypothesis testing, the test statistic (e.g. *F*, *t*, *r*) with confidence intervals, effect sizes, degrees of freedom and *P* value noted<br>*Give P values as exact values whenever suitable.*
☒	☐	For Bayesian analysis, information on the choice of priors and Markov chain Monte Carlo settings
☒	☐	For hierarchical and complex designs, identification of the appropriate level for tests and full reporting of outcomes
☒	☐	Estimates of effect sizes (e.g. Cohen's *d*, Pearson's *r*), indicating how they were calculated

Our web collection on statistics for biologists contains articles on many of the points above.

Software and code

Policy information about availability of computer code

Data collection	A commercially available Nikon Ti-E was used to obtain microscopy data, and a commercially available Beckman Coulter, CytoFlex S was used for cell count acquisition.
Data analysis	Data handling, statistics and data presentation was carried out using Microsoft Excel (Version 2412 Build 16.0.18324.20092) and Graphpad Prism 10.4.1 (GraphPad Software, La Jolla, CA, USA). Data tracking was performed with the publicly available TrackPy v.0.6.1 published on https://doi.org/10.5281/zenodo.7670439 (2023).

For manuscripts utilizing custom algorithms or software that are central to the research but not yet described in published literature, software must be made available to editors and reviewers. We strongly encourage code deposition in a community repository (e.g. GitHub). See the Nature Portfolio guidelines for submitting code & software for further information.

Data

Policy information about availability of data

All manuscripts must include a data availability statement. This statement should provide the following information, where applicable:

- Accession codes, unique identifiers, or web links for publicly available datasets
- A description of any restrictions on data availability
- For clinical datasets or third party data, please ensure that the statement adheres to our policy

Data files and codes used during the current study have been submitted are publicly available at the ETH Research Collection which can be accessed at this DOI: https://doi.org/10.3929/ethz-b-000745131.

Research involving human participants, their data, or biological material

Policy information about studies with human participants or human data. See also policy information about sex, gender (identity/presentation), and sexual orientation and race, ethnicity and racism.

Reporting on sex and gender	Not applicable for this study.
Reporting on race, ethnicity, or other socially relevant groupings	Not applicable for this study.
Population characteristics	Not applicable for this study.
Recruitment	Not applicable for this study.
Ethics oversight	Not applicable for this study.

Note that full information on the approval of the study protocol must also be provided in the manuscript.

Field-specific reporting

Please select the one below that is the best fit for your research. If you are not sure, read the appropriate sections before making your selection.

☐ Life sciences ☐ Behavioural & social sciences ☒ Ecological, evolutionary & environmental sciences

For a reference copy of the document with all sections, see nature.com/documents/nr-reporting-summary-flat.pdf

Ecological, evolutionary & environmental sciences study design

All studies must disclose on these points even when the disclosure is negative.

Study description	A study using C. braarudii to investigate the long-standing hypothesis that the calcium carbonate shell covering most coccolithophores during their diploid life stage can protect them against bacterial attack.
Research sample	No statistical method was employed to pre-determine the sample size. For tracking experiments bulk counts of 20,000 cells were used, with a minimum of three biological replicates per analysis.
Sampling strategy	No specific sampling strategy was employed.
Data collection	Data was collected daily (or every other day) for flow cytometry data, agitating algal cultures to resuspend the cells. For timelapse microscopy experiments, an image was taken every hour automatically. All data was collected by the same person and analyzed using the same machines.
Timing and spatial scale	Lab-based experimentation was carried out. Experiments were tracked across multiple days, for up to ten days.
Data exclusions	No data was excluded.
Reproducibility	All data sets contained at least three biological replicates coming from discrete cultures grown independently from each other.
Randomization	Not applicable to this study.
Blinding	Not applicable to this study.

Did the study involve field work? ☐ Yes ☒ No

Reporting for specific materials, systems and methods

We require information from authors about some types of materials, experimental systems and methods used in many studies. Here, indicate whether each material, system or method listed is relevant to your study. If you are not sure if a list item applies to your research, read the appropriate section before selecting a response.

Materials & experimental systems

n/a	Involved in the study
☒	☐ Antibodies
☐	☒ Eukaryotic cell lines
☒	☐ Palaeontology and archaeology
☒	☐ Animals and other organisms
☒	☐ Clinical data
☒	☐ Dual use research of concern
☒	☐ Plants

Methods

n/a	Involved in the study
☒	☐ ChIP-seq
☐	☒ Flow cytometry
☒	☐ MRI-based neuroimaging

Eukaryotic cell lines

Policy information about cell lines and Sex and Gender in Research

Cell line source(s)	RCC1200 was purchased from the Roscoff Culture Collection.
Authentication	No authentication procedure was used, however, visual appearance was monitored, and all cell lines were purchased directly from the algal depositories for this paper.
Mycoplasma contamination	Cells lines were not tested for mycoplasma contamination.
Commonly misidentified lines (See ICLAC register)	Not applicable to this study.

Plants

Seed stocks	Not applicable to this study.
Novel plant genotypes	Not applicable to this study.
Authentication	Not applicable to this study.

Flow Cytometry

Plots

Confirm that:

☒ The axis labels state the marker and fluorochrome used (e.g. CD4-FITC).

☒ The axis scales are clearly visible. Include numbers along axes only for bottom left plot of group (a 'group' is an analysis of identical markers).

☒ All plots are contour plots with outliers or pseudocolor plots.

☒ A numerical value for number of cells or percentage (with statistics) is provided.

Methodology

Sample preparation	Algae samples did not require preparation and bacteria samples were diluted to (<10^6 cells/mL) stained with SYBR green (1:10,000 final dilution, ThermoFischer) and incubated in the dark for 10 minutes prior to analysis.
Instrument	CytoFLEX S (Beckman Coulter, USA)
Software	CytExpert Version 2.4

Cell population abundance	Cell counts measured directly with the machine were always kept below x10^6 cells/mL to prevent clogging. Lowest cell counts measured were in the 10^4 cells/mL range.
Gating strategy	Gates were determined by looking at untreated controls and decalcified cultures as well as background control pure seawater samples. The untreated and decalcified culture samples were confirmed using microscopy. Figures showing the gating strategy used are provided in the SI.

☒ Tick this box to confirm that a figure exemplifying the gating strategy is provided in the Supplementary Information.
